# Genome-Wide Analysis of Ascorbic Acid Metabolism Related Genes in *Fragaria* × *ananassa* and Its Expression Pattern Analysis in Strawberry Fruits

**DOI:** 10.3389/fpls.2022.954505

**Published:** 2022-07-06

**Authors:** Huabo Liu, Lingzhi Wei, Yang Ni, Linlin Chang, Jing Dong, Chuanfei Zhong, Rui Sun, Shuangtao Li, Rong Xiong, Guixia Wang, Jian Sun, Yuntao Zhang, Yongshun Gao

**Affiliations:** ^1^Institute of Forestry and Pomology, Beijing Academy of Forestry and Pomology Sciences, Beijing, China; ^2^Key Laboratory of Biology and Genetic Improvement of Horticultural Crops (North China), Ministry of Agriculture and Rural Affairs, Beijing, China; ^3^Beijing Engineering Research Center for Strawberry, Beijing, China; ^4^Inspection and Testing Laboratory of Fruits and Nursery Stocks (Beijing), Ministry of Agriculture and Rural Affairs, Beijing, China

**Keywords:** *Fragaria* × *ananassa*, ascorbic acid biosynthesis, genome-wide analysis, AKR, expression pattern

## Abstract

Ascorbic acid (AsA) is an important antioxidant for scavenging reactive oxygen species and it is essential for human health. Strawberry (*Fragaria* × *ananassa*) fruits are rich in AsA. In recent years, strawberry has been regarded as a model for non-climacteric fruit ripening. However, in contrast to climacteric fruits, such as tomato, the regulatory mechanism of AsA accumulation in strawberry fruits remains largely unknown. In this study, we first identified 125 AsA metabolism-related genes from the cultivated strawberry “Camarosa” genome. The expression pattern analysis using an available RNA-seq data showed that the AsA biosynthetic-related genes in the D-mannose/L-galactose pathway were downregulated remarkably during fruit ripening which was opposite to the increasing AsA content in fruits. The D-galacturonate reductase gene (*GalUR*) in the D-Galacturonic acid pathway was extremely upregulated in strawberry receptacles during fruit ripening. The *FaGalUR* gene above belongs to the aldo-keto reductases (AKR) superfamily and has been proposed to participate in AsA biosynthesis in strawberry fruits. To explore whether there are other genes in the AKR superfamily involved in regulating AsA accumulation during strawberry fruit ripening, we further implemented a genome-wide analysis of the AKR superfamily using the octoploid strawberry genome. A total of 80 *FaAKR* genes were identified from the genome and divided into 20 subgroups based on phylogenetic analysis. These *FaAKR* genes were unevenly distributed on 23 chromosomes. Among them, nine genes showed increased expression in receptacles as the fruit ripened, and notably, *FaAKR23* was the most dramatically upregulated *FaAKR* gene in receptacles. Compared with fruits at green stage, its expression level increased by 142-fold at red stage. The qRT-PCR results supported that the expression of *FaAKR23* was increased significantly during fruit ripening. In particular, the *FaAKR23* was the only *FaAKR* gene that was significantly upregulated by abscisic acid (ABA) and suppressed by nordihydroguaiaretic acid (NDGA, an ABA biosynthesis blocker), indicating *FaAKR23* might play important roles in ABA-mediated strawberry fruit ripening. In a word, our study provides useful information on the AsA metabolism during strawberry fruit ripening and will help understand the mechanism of AsA accumulation in strawberry fruits.

## Introduction

L-Ascorbic acid (AsA, also known as vitamin C) is a water-soluble antioxidant in plants that plays an important role in various plant physiological processes, including photosynthesis, electron transport, plant growth, and resistance to environmental stresses (Akram et al., 2017; Fenech et al., 2019). AsA is involved in the ascorbate–glutathione cycle, which is an effective metabolic cycle for hydrogen peroxide and excess energy dissipation (Akram et al., 2017). For humans, AsA is also an indispensable nutrient, which plays an important role in protecting human health and disease prevention. However, human beings and a few other animal species have lost the capability to synthesize AsA by themselves due to a mutation in the last enzyme required in the AsA biosynthetic pathway (Chatterjee, 1973). Thus, humans have to acquire AsA from dietary sources such as fresh fruits and vegetables (Ishikawa et al., 2018).

Over the past decades, four AsA biosynthetic pathways in plants have been proposed, including D-mannose/L-galactose (D-Man/L-Gal) pathway, D-galacturonate (D-GalUA) pathway, D-glucuronate pathway, and L-Gulose pathway (Ishikawa et al., 2018). Among these pathways, the D-Man/L-Gal pathway is responsible for the major biosynthetic pathway of AsA in higher plants (Wheeler et al., 1998). This pathway catalyzes the conversion of D-Fructose 6-P to AsA, which consists of eight reaction steps catalyzed by phosphomannose isomerase (PMI), phosphomannose mutase (PMM), GDP-D-mannose pyrophosphorylase (GMP), GDP-D-mannose-3’,5’-epimerase (GME), GDP-L-galactose phosphorylase (GGP), L-galactose-1-phosphate phosphatase (GPP), L-galactose dehydrogenase (L-GalDH), and L-galactono-1,4-lactone dehydrogenase (L-GalLDH) (Ishikawa et al., 2018). All of the genes of enzymes described above in the D-Man/L-Gal pathway have been identified in *Arabidopsis*. In plants, the D-GalUA pathway, D-glucuronate pathway, and L-Gulose pathway were proposed as alternative AsA biosynthesis pathways, and the prevalence of these pathways in different tissues or developmental stages is largely unknown. However, in the D-GalUA pathway, although the D-galacturonate reductase (GalUR) was first isolated from strawberries, the GalUR from other plants has not been identified and studied in depth (Agius et al., 2003; Ishikawa et al., 2018). Moreover, the aldonolactonase, an important characteristic enzyme in the D-GalUA pathway, which catalyzes L-galactonic acid to L-galactono-1,4-lactone, has not been identified in any plant (Sodeyama et al., 2021). Several previous studies suggested that the D-GalUA pathway contributed to AsA accumulation in ripening fruits (Cruz-Rus et al., 2010, 2011; Badejo et al., 2012). This hypothesis suggests that D-galacturonate could be supplied by the breakdown of pectin in the cell wall during the softening process of fruits, and explains why the D-GalUA pathway seems to be activated at the latter stages of fruit ripening (Badejo et al., 2012; Ishikawa et al., 2018). However, there is a lack of genetic evidence to support this hypothesis to date. In the glucuronate pathway, the precursor of D-glucuronate derives either from glucose or myo-inositol, and the latter could be converted to D-glucuronate by myo-inositol oxygenase (MIOX), so this pathway is also called as Myo-inositol pathway (Lorence et al., 2004). However, genetic evidence for this pathway contributing to AsA biosynthesis in plants remains limited (Ishikawa et al., 2018; Ivanov Kavkova et al., 2019). The L-gulose pathway is a branch of the D-Man/L-Gal pathway, beginning with GDP-D-mannose and converting to AsA via L-gulose. At present, the catalytic enzymes in this pathway have not yet been identified in any plant (Ishikawa et al., 2018). Therefore, whether this pathway contributes to AsA accumulation is also unclear. In addition to AsA biosynthesis, AsA recycling also contributes to AsA content in plants (Chen et al., 2003; Wang et al., 2010). Ascorbate peroxidases (APX) catalyze the reduction of H_2_O_2_ into the water using two AsA molecules, resulting in the formation of two molecules of monodehydroascorbate (MDHA). MDHA could be reduced back to AsA by monodehydroascorbate reductases (MDHAR). Meanwhile, two MDHAs are spontaneously disproportionated to AsA and dehydroascorbate (DHA). DHA is reduced back to AsA by dehydroascorbate reductases (DHAR) (Maruta and Ishikawa, 2018).

The cultivated strawberry (*Fragaria* × *ananassa* Duch.) is one of the most popular fruits throughout the world, which has excellent nutritional and commercial value due to its numerous nutrients and unique flavor (Liu et al., 2021). Strawberry fruits are rich in AsA, with an average level of 58.8 mg/100 g FW (US Department of Agriculture [USDA], 2018), which is regarded as high AsA fruits. In addition, the AsA concentration in strawberry fruits has also been reported to continuously increase during fruit development and ripening (Agius et al., 2003; Wang et al., 2021; Siebeneichler et al., 2022). However, AsA content varies widely among different strawberry varieties or species (Cruz-Rus et al., 2011; Fenech et al., 2019). Thus, strawberry fruits were very suitable for mining the key genes of AsA accumulation. Significantly, among the genes of AsA biosynthetic pathways, *FaGalUR* showed a dramatic increase (Cruz-Rus et al., 2011). Overexpression of *FaGalUR* in *Arabidopsis* (Agius et al., 2003), potato (Hemavathi et al., 2009), and tomato (Cai et al., 2014; Lim et al., 2016) led to a multifold increase of AsA content in the leaves and fruits of transgenic plants, respectively. Meanwhile, the transgenic plants also enhanced their tolerance to abiotic stresses (Cai et al., 2014; Lim et al., 2016). These results indicate that the *FaGalUR* plays significant roles in the AsA biosynthesis and can be used as a target to be genomically modified to improve the quality of strawberry fruits. However, to date, the D-galacturonate pathway toward AsA biosynthesis is still considerably controversial (Broad et al., 2020; Mellidou et al., 2021). So far, there are no studies of knockout *FaGalUR* to evaluate the relative contribution of the D-GalUA pathway toward AsA biosynthesis in strawberries (Broad et al., 2020). GalUR is a member of the aldo-keto reductases (AKR) superfamily, which comprises a diverse family of homologous genes. However, apart from *FaGalUR*, no AKR genes have been found to contribute to AsA biosynthesis in other plant species. The availability of octoploid strawberry genome sequence and several diploid strawberry genome sequences provide us a chance to identify AsA metabolism-related genes and some important gene families such as the AKR superfamily and gain further insight into the mechanism of AsA biosynthesis in strawberry fruits. At present, 11 GMP homologous genes in the octoploid strawberry genome (Lin et al., 2021) and 33 AKR gene family members in *Fragaria vesca* (Duan et al., 2020) have been identified. However, in octoploid strawberry, there is neither available information on the entire AsA metabolism-related genes nor the AKR superfamily involved in strawberry fruit ripening. In this study, we conducted a genome-wide identification of AsA metabolism-related genes and AKR superfamily in the octoploid strawberry genome “Camarosa”. We analyzed their expression pattern in strawberry fruit ripening and response to ABA treatment according to two available open-published transcriptome data. A total of 125 AsA metabolism-related genes and 80 *FaAKR* genes were identified in octoploid strawberry. Finally, from green stage to red stage, the *FaAKR23* showed the highest transcript level among all nine upregulated *FaAKR* genes in strawberry fruits, which was significantly upregulated by abscisic acid (ABA) and suppressed by nordihydroguaiaretic acid (NDGA, an ABA biosynthesis blocker), suggesting that it might play important roles during strawberry fruit ripening. The results of this work provide new insights into the AsA biosynthesis in strawberry fruits and the *FaAKR23* might be a candidate gene for further functional study to improve the quality of strawberry fruits.

## Materials and Methods

### Genome-Wide Identification of Ascorbic Acid Metabolism-Related Genes and FaAKR in Octoploid Strawberry

The deduced protein sequences of AsA metabolism-related genes in *Arabidopsi*s were retrieved and downloaded from the TAIR database,^1^ and then performed a local BLASTP search against the cultivated octoploid strawberry genome ‘‘Camarosa’’, which was downloaded from the GDR database.^2^ A hidden Markov Model (HMM) profile of Aldo-ket red (PF00248) was downloaded from the Pfam database^3^ and used to identify all FaAKR sequences from the octoploid strawberry genome by Hmmer3.0 software (Mistry et al., 2013) with e-value 1e-5. All candidate sequences were further examined and validated by CDD,^4^ Pfamscan,^5^ and SMART.^6^ The characteristic of FaAKR proteins, including protein length, isoelectric point (PI), molecular weight (MW), and grand average of hydropathicity (GRAVY) were predicted by ExPASy.^7^ All *FaAKR* genes were mapped to the chromosomes using MapChart (Voorrips, 2002) and renamed according to their distribution order on chromosomes.

### Phylogenetic Analysis and Collinearity Analysis of FaAKR

The AKR sequences of *F.* × *ananassa* and *F. vesca* were aligned by MUSCLE. A maximum likelihood (ML) phylogenetic tree was constructed using MEGA-X software (Kumar et al., 2018) with a bootstrap value of 1,000. The collinearity of *FaAKR* genes was determined using the Multiple Collinearity Scan toolkit MCScanX (Wang et al., 2012) and visualized using Circos (Krzywinski et al., 2009).

### Promoter Sequence Analysis of *FaAKR* Genes

The 2,000 bp sequence upstream from the start codon (ATG) of *FaAKR* genes was extracted from the octoploid strawberry genome and used as promoter regions. The *cis*-elements of *FaAKR* genes were identified by PlantCARE.^8^

### Transcriptome Analysis

The transcriptome data from fruits at four ripening stages, leaves, and roots from *F.* × *ananassa* cv. “Camarosa” (PRJEB12420) were downloaded from the NCBI database and used to analyze the expression pattern of AsA metabolism-related genes and *FaAKR* genes at different fruit ripening stages. The transcriptome data from *F.* × *ananassa* cv. “Toyonoka” with exogenous ABA and NDGA treatment (PRJNA338879) were downloaded and used to analyze the gene expression pattern in response to plant phytohormone. The raw RNA-seq data were reanalyzed according to the octoploid strawberry genomic data (v1.0.a1). The TPM (transcripts per million) values were used to measure transcript expression levels.

### Plant Materials, RNA Extraction, and qRT-PCR

The cultivated strawberry (*F.* × *ananassa* cv. “Sweet Charlie”) was grown in a greenhouse at the Beijing Academy of Forestry and Pomology Sciences, Beijing, China. The strawberry fruits were sampled from five ripening stages, including small green (SG), big green (BG), white (W), pink (P), and red (R). All samples were immediately frozen in liquid nitrogen and stored at −80°C for further analysis. Three biological replicates of five fruit each were used. Total RNA was extracted from receptacles after the removal of achenes. The quality and concentration of RNA were verified using agarose gel electrophoresis and a a Nanodrop 2000 spectrophotometer. The qRT-PCR was performed using TB Green™ Premix Ex Taq™ II on a CFX96™ Real-Time System (Bio-Red). The relative expression of genes was calculated by the 2^–ΔΔ*Ct*^ method using *FaCHP1* as the internal control (Clancy et al., 2013). Three technical replicates were done. The primers used are listed in Supplementary Table 1.

## Results

### Identification of Ascorbic Acid Metabolism-Related Genes in Octoploid Strawberry

The AsA metabolism-related protein sequences from the *Arabidopsis* Information Resource (TAIR) database were downloaded and used to identify candidate genes in the octoploid strawberry genome “Camarosa”. A total of 125 AsA metabolism-related genes were identified in strawberry (Table 1). Among them, 64 genes belong to the biosynthesis pathway and 61 genes belong to the recycling pathway.

**TABLE 1:** Number of predicted genes encoding enzymes of ascorbic acid (AsA) biosynthesis and regeneration in *Arabidopsis thaliana* and *Fragaria* spp.

Gene	Description	*Arabidopsis thaliana*	*Fragaria vesca*	*Fragaria* × *ananassa*
PMI	Mannose-6-phosphate isomerase	2	2	7
PMM	Phosphomannomutase	1	1	4
GMP	GDP-D-mannose pyrophosphorylase	1	1	5
GME	GDP-D-mannose-3,5-epimerase	1	1	5
GGP	GDP-L-galactose phosphorylase	2	3	12
GPP	L-galactose-1-phosphate phosphatase	1	1	3
GalDH	L-galactose dehydrogenase	1	1	4
GalLDH	L-galactono-1,4-lactone dehydrogenase	1	1	4
GalUR	D-galacturonate reductase	0	1	4
MIOX	Myo-inositol oxygenase	4	5	16
AO	L-ascorbate oxidase	3	3	9
APX	L-ascorbate peroxidase	7	8	24
DHAR	Dehydroascorbate reductase	4	4	12
MDHAR	Monodehydroascorbate reductase	4	4	16

### Expression Pattern Analysis of Ascorbic Acid Metabolism-Related Genes in Strawberry

**FIGURE 1:**
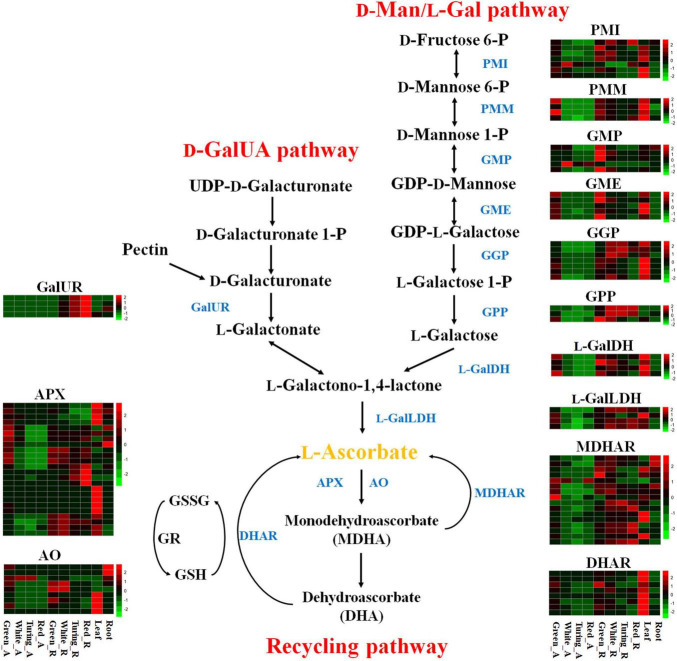
Transcript abundance of 100 genes involved in ascorbic acid (AsA) metabolism in octoploid strawberry “Camarosa”. Genes that were not expressed or expressed at an extremely low level were not shown. Enzymes are displayed in dark blue: PMI, phosphomannose isomerase; PMM, phosphomannose mutase; GMP, GDP-D-mannose pyrophosphorylase; GME, GDP-D-mannose-3′,5′-epimerase; GGP, GDP-L-galactose phosphorylase; GPP, L-galactose-1-phosphate phosphatase; L-GalDH, L-galactose dehydrogenase; L-GalLDH, L-galactono-1,4-lactone dehydrogenase; GalUR, D-galacturonate reductase; APX, L-ascorbate peroxidase; AO, L-ascorbate oxidase; DHAR, dehydroascorbate reductase; MDHAR, monodehydroascorbate reductase; Green_A, green achene; White_A, white achene; Turning_A, turning achene; Red_A, red achene; Green_R, green receptacle; White_R, white receptacle; Turning_R, turning receptacle; Red_R, red receptacle.

**FIGURE 2:**
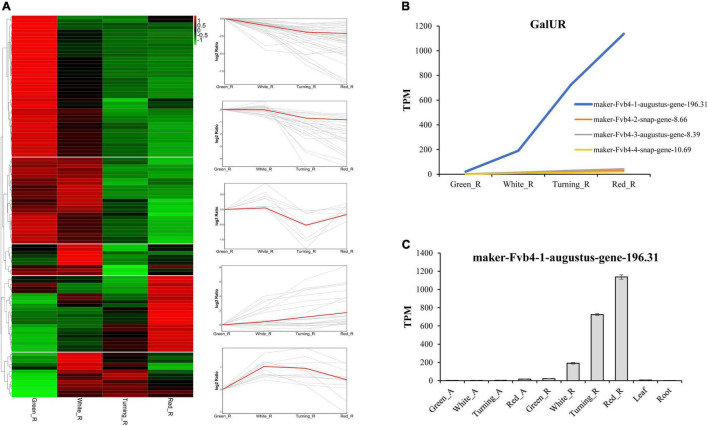
Expression pattern analysis of ascorbic acid (AsA) metabolism-related genes in four ripening stages of octoploid strawberry “Camarosa”. **(A)** The heat map and trend analysis of AsA metabolism-related genes in receptacles. **(B)** Expression pattern of four GalUR homologs genes in receptacles. **(C)** Expression pattern of *FaGalUR* gene (maker-Fvb4-1-augustus-gene-196.31) in achenes and receptacles at four ripening stages, leaves, and roots. Green_A, green achene; White_A, white achene; Turning_A, turning achene; Red_A, red achene; Green_R, green receptacle; White_R, white receptacle; Turning_R, turning receptacle; Red_R, red receptacle.

To analyze the expression pattern of AsA metabolism-related genes during strawberry fruit ripening, we used transcriptome data from receptacles and achenes at four ripening stages, leaves, and roots from *F.* × *ananassa* cv. “Camarosa” (Sánchez-Sevilla et al., 2017). The raw RNA-seq data were reanalyzed according to the octoploid strawberry genomic data (Edger et al., 2019). The expression data showed that most AsA biosynthetic genes in the D-Man/L-Gal pathway were downregulated during strawberry fruit ripening (Figure 1). For example, the GMP and GME were decreased by 33.21∼81.42% and 66.76∼96.00% in receptacles from green stage to red stage, respectively (Supplementary Table 2). Thirteen genes were upregulated at first but then decreased during fruit ripening, in which GGP and GPP were the fourth and third steps from last in the D-Man/L-Gal pathway, indicating that the contribution of AsA accumulation from the D-Man/L-Gal pathway was gradually reduced from white stage to red stage (Figure 2). A total of 22 genes showed increased expression during ripening, and from the above, 12 genes belonged to the recycling pathway, including eight APX genes and four MDHAR genes, suggesting the potential significance of regeneration for AsA accumulation in strawberry fruits. The *FaGalUR* gene (maker-Fvb4-1-augustus-gene-196.31) was the most extremely upregulated in receptacles during fruit ripening, but it was hardly expressed in achenes, leaves, and roots (Figures 2B,C). Its expression levels in receptacles increased by 54-fold from green stage to red stage, which suggested that *FaGalUR* might play an important role in the AsA accumulation of strawberry fruit.

Previous studies found that ABA is vital for strawberry fruit ripening and the AsA content in ABA-treated receptacles significantly increased compared with the control samples (Li et al., 2015, 2019), but the mechanism of AsA accumulation in response to ABA is still unknown. To investigate the expression changes of AsA metabolism-related genes in response to ABA, we further performed a reanalysis using the transcriptome data from *F.* × *ananassa* cv. “Toyonoka” with exogenous ABA or NDGA treatment (Li et al., 2019). The results showed that most of the AsA metabolism-related genes (78.90%) were downregulated, whereas, 12.84% of genes showed upregulated expression on the 5th day, and then followed by downregulation on the 8th day, only nine genes were upregulated by ABA treatment (Supplementary Table 3). Eight out of nine upregulated genes belonged to the recycling pathway, in which six genes were APX genes, indicating that ABA treatment accelerated the consumption of reduced AsA with the oxidation to MDHA and DHA. Significantly, the *FaGalUR* were downregulated by ABA treatment, but not upregulated under normal ripening conditions (Figure 2). These results could not explain why exogenous ABA treatment can promote AsA accumulation in strawberry fruit. Thus, it is speculated that there may be unidentified genes or another unknown mechanism that could promote AsA accumulation by ABA treatment.

### Identification and Characteristics of *FaAKR* Genes in Octoploid Strawberry

Although the D-galacturonate pathway has been proved to contribute to AsA accumulation in strawberry fruits (Agius et al., 2003), the information of this pathway is still limited. Especially, many genes encoding enzymes involved in the pathway have not yet been identified in any plants (Ishikawa et al., 2018). GalUR is classified as the AKR superfamily member which is involved in diverse plant metabolic processes and stress defense (Sengupta et al., 2015). Thus, we continued to conduct a genome-wide analysis of the AKR superfamily in the octoploid strawberry genome to identify potential genes which may play important roles in AsA biosynthesis during strawberry fruit ripening.

**FIGURE 3:**
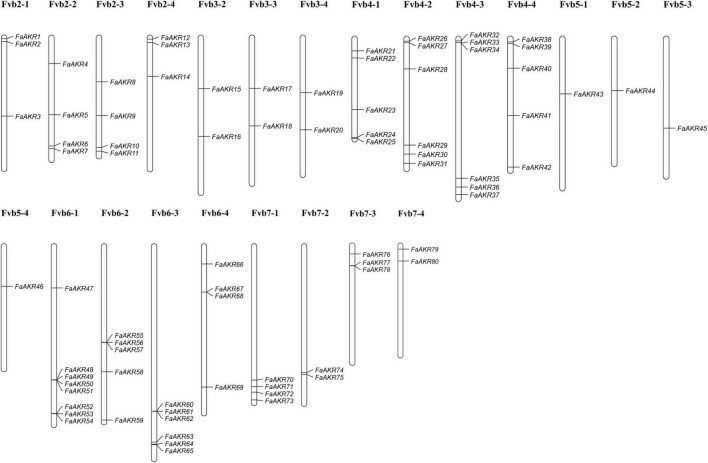
Chromosomal distribution of *FaAKR* genes in *Fragaria* × *ananassa*. The putative *FaAKR* genes were renamed from *FaAKR1* to *FaAKR80*, based on their placement on chromosomes.

To identify homologous AKR genes in *F.* × *ananassa*, the Hidden Markov Model (HMM) profiles of Aldo-ket red (PF00248) were downloaded and used to search against the octoploid strawberry genome. A total of 80 *FaAKR* genes were identified in the *F.* × *ananassa* genome, and we renamed them as *FaAKR1* to *FaAKR80* according to their distribution order on chromosomes, and the related information of gene ID and gene name were listed in Supplementary Table 4. As shown, the AKR genes were unevenly distributed on 23 chromosomes (Figure 3). Specifically, there were no AKR genes located on chromosomes Fvb1-1 ∼ Fvb1-4, and Fvb3-1. Moreover, more than half of the *FaAKR* genes are located on chromosomes Fvb4-1 ∼ Fvb4-4 and Fvb6-1 ∼ Fvb6-4, including 22 and 23 genes, respectively, among which Fvb6-1, containing eight genes, exhibited the highest number of AKR genes in the subgenome level of the octoploid strawberry genome. The *FaAKR43* ∼ *FaAKR46* were located on chromosome Fvb5-1 ∼ Fvb5-4, with only one AKR gene in each subgenome, respectively. Among these genes, *FaAKR24*, *FaAKR27*, *FaAKR33*, and *FaAKR39* were the homologous gene of *FaGalUR* and *FaAKR2*, *FaAKR6*, *FaAKR10*, and *FaAKR13* were homologous gene of *FaGalDH*.

**FIGURE 4:**
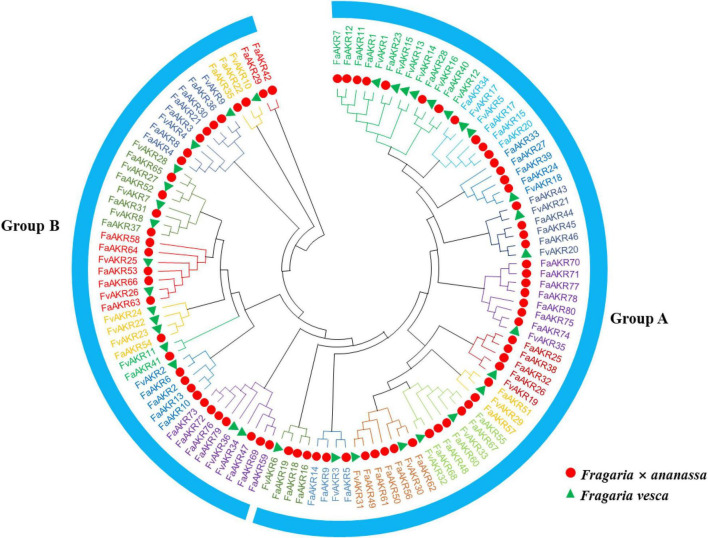
Phylogenetic relationships of AKR proteins between *Fragaria* × *ananassa* and *Fragaria vesca*. The red circles and green triangles represent genes from *F. × ananassa* and *F. vesca*, respectively. All 20 subfamilies of AKRs were represented by different colors.

To analyze the evolutionary relationships of the AKR gene family members, a maximum likelihood (ML) tree containing 80 FaAKR proteins and 36 FvAKR proteins was constructed using MEGA-X. The results showed that all 116 AKR proteins were divided into two distinct groups (groups A and B) and 20 subgroups (Figure 4). Group A consisted of 42 FaAKR members and 18 FvAKR members and group B consisted of 38 FaAKR members and 18 FvAKR members (Figure 4). Groups A and B showed similar numbers of members. Interestingly, the homologous genes of *FaGalUR* (*FaAKR24*, *FaAKR27*, *FaAKR33*, and *FaAKR39*) and *FaGalDH* (*FaAKR2*, *FaAKR6*, *FaAKR10*, and *FaAKR13*) were located in different groups, suggesting a diverse function in two groups.

**FIGURE 5:**
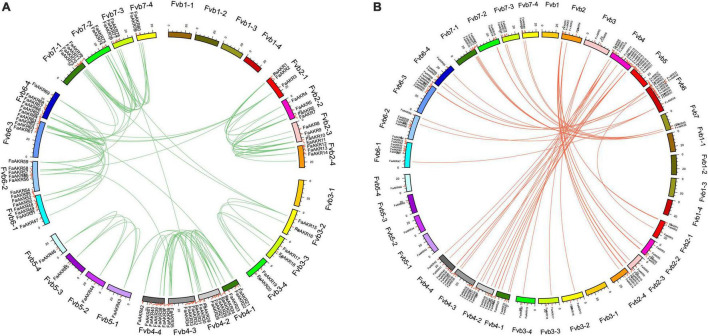
Collinearity analyses of AKR genes. **(A)** Collinearity analyses of AKR genes in *Fragaria* × *ananassa*; **(B)** interspecific collinearity analyses of AKR genes between *F.* × *ananassa* and *Fragaria vesca* genomes. Outer boxes represented chromosomes. Lines in boxes indicated the location of AKR genes in each chromosome. Gene pairs with a synthetic relationship are joined by colored lines.

To elucidate the evolution and expansion of the *FaAKR* superfamily in *Fragaria*, we investigated the collinearity of AKR genes in the *F.* × *ananassa* genome. There were 114 pairs of AKR genes showing a synteny relationship among the octoploid strawberry genome, of which 84.21% of the genes were collinear with several genes in the subgenome of the same chromosome (Figure 5A). However, 18 pairs (15.79%) showed segmental duplication among different chromosomes, suggesting that these genes might arise from the whole genome duplication events. The interspecific synteny among *F.* × *ananassa* and *F. vesca* genomes was also analyzed. The results showed that there were 55 pairs of orthologous genes between *F.* × *ananassa* and *F. vesca* (Figure 5B).

The *cis*-elements play important roles in the regulation of gene expression. In this study, the upstream 2,000 bp nucleotide sequences from 80 *FaAKR* genes were extracted and used to predict *cis*-acting regulatory elements. The results showed that *cis*-elements involved in light responsiveness, hormones responsiveness, and anaerobic induction broadly existed in promoter regions. Remarkably, there were 460 hormones responsiveness elements, including 165 abscisic acid responsiveness elements (ABREs), 149 MeJA-responsiveness elements (CGTCA-motif), 60 gibberellin-responsive elements (GARE, P-box, and TATC-box), 49 auxin-responsive elements (TGA and AuxRR-core), and 37 salicylic acid responsiveness element (SARE and TCA-element), suggesting that the expression of *FaAKR* genes might be regulated by diverse plant hormones (Supplementary Table 5). In addition, anaerobic induction (ARE) and light responsiveness (G-box) elements were found in most of the *FaAKR* genes, while a *cis*-acting regulatory element root-specific motif I only existed in the promoter of *FaAKR15*.

### Expression Pattern Analysis of *FaAKR* Genes in Strawberry

**FIGURE 6:**
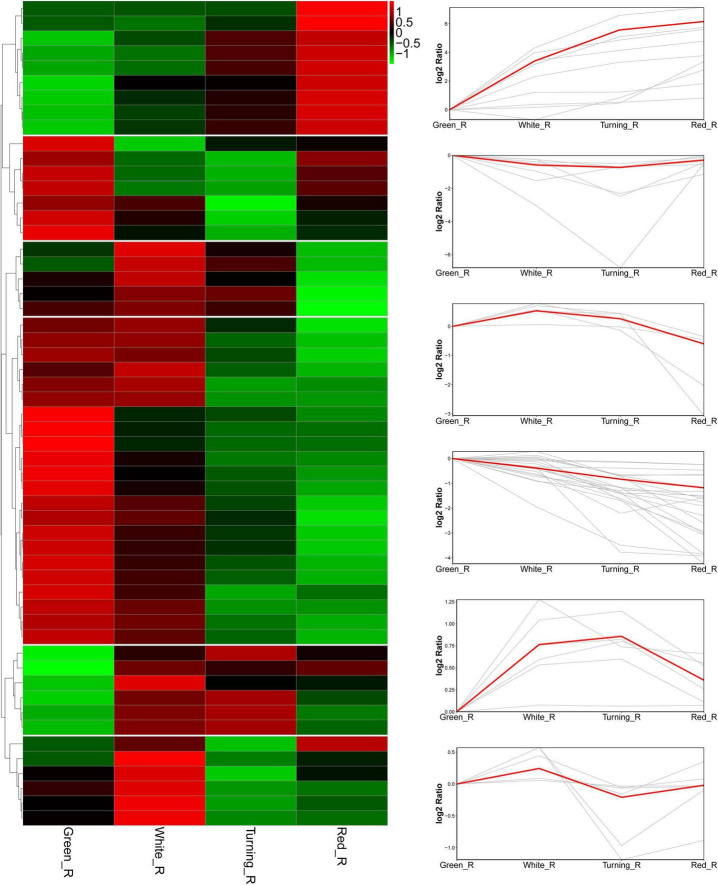
Heat map and trend analysis of *FaAKR* genes in receptacles at four ripening stages of octoploid strawberry “Camarosa”. Green_R, green receptacle; White_R, white receptacle; Turning_R, turning receptacle; Red_R, red receptacle.

To understand the potential functions of *FaAKR* genes, the transcriptome data from *F.* × *ananassa* cv. “Camarosa” (Sánchez-Sevilla et al., 2017) were further used to analyze the expression pattern of *FaAKR* genes. A total of 25 genes were not expressed or expressed at a very low level in receptacles. The trend analysis of the remaining genes showed that the *FaAKR* superfamily has six different expression trends during fruit ripening (Figure 6 and Supplementary Table 6). Nine genes (*FaAKR9*, *FaAKR23*, *FaAKR24*, *FaAKR27*, *FaAKR33*, *FaAKR39*, *FaAKR41*, *FaAKR49*, and *FaAKR53*) were gradually upregulated during fruit ripening, 22 genes were downregulated gradually, while five genes (*FaAKR3*, *FaAKR14*, *FaAKR52*, *FaAKR59*, and *FaAKR61*) were upregulated at first but then downregulated and seven genes (*FaAKR2*, *FaAKR4*, *FaAKR25*, *FaAKR44*, *FaAKR50*, *FaAKR63*, and *FaAKR65*) were downregulated at first but then upregulated (Supplementary Table 6).

**FIGURE 7:**
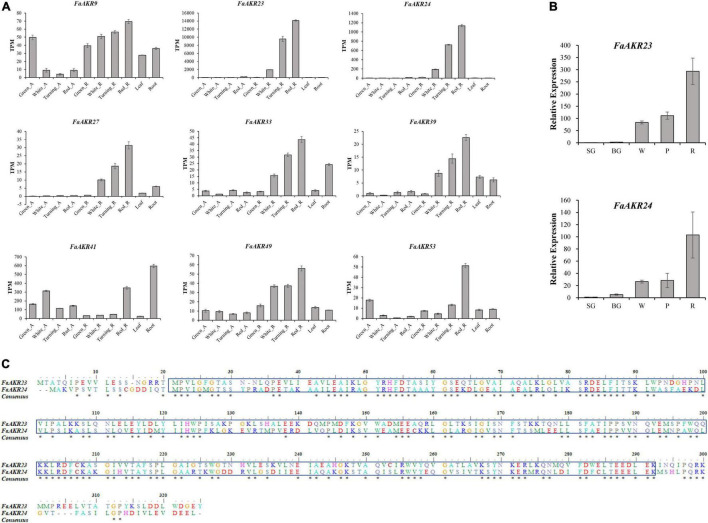
Expression pattern of upregulated *FaAKR* genes in octoploid strawberry. **(A)** Expression pattern of nine upregulated *FaAKR* genes in achenes and receptacles at four ripening stages, leaves, and roots using RNA-seq data. **(B)** Relative expression levels of *FaAKR23* and *FaAKR24* at five ripening stages in *Fragaria ananassa* cv. “Sweet Charlie” by qRT-PCR. Green_A, green achene; White_A, white achene; Turning_A, turning achene; Red_A, red achene; Green_R, green receptacle; White_R, white receptacle; Turning_R, turning receptacle; Red_R, red receptacle; SG, small green fruit; BG, big green fruit; W, white fruit; P, pink fruit; R, red fruit. **(C)** Sequence alignment of FaAKR23 and FaAKR24. The box indicates the Aldo-ket red domain; the conserved amino acid residues are labeled with an asterisk.

Among nine upregulated *FaAKR* genes, the level of *FaAKR23* transcript was the highest in receptacles and its expression levels increased by 142-fold from green stage to red stage, followed by the *FaAKR24* which was the homologous gene of *FaGalUR* (Figure 7A). Furthermore, the expression levels of *FaAKR23* and *FaAKR24* were higher in receptacles than in the othre tissues. The qRT-PCR results supported that the expression level of *FaAKR23* and *FaAKR24* increased gradually along with the ripening of fruits (Figure 7B). Importantly, the results of sequence alignment (Figure 7C) and phylogenetic analyses (Figure 4) show that *FaAKR23* and *FaAKR24* have a close relationship, suggesting they might have similar functions and be crucial in strawberry fruit ripening. It is noticed that some genes were strongly expressed not only in receptacles but also in other tissues, for instance, the expression of *FaAKR33* and *FaAKR41* was strong in root and *FaAKR53* in achenes at the green stage of fruit ripening, while *FaAKR9* exhibited strong expression in leaves, root, and achenes at the green stage of fruit ripening, implying their important roles in different tissues and fruit ripening (Figure 7A and Supplementary Table 6).

**FIGURE 8:**
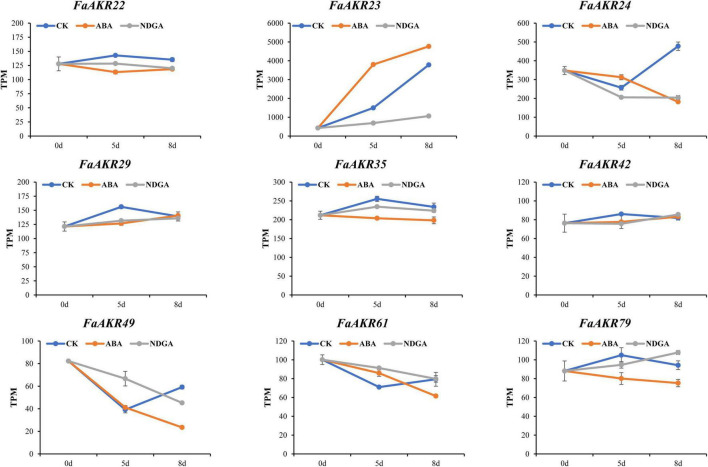
Trend analysis of *FaAKR* genes in response to ABA and NDGA treatment. CK, ABA, and NDGA represent control, abscisic acid, and nordihydroguaiaretic acid-treated fruits, respectively. The x-axis represents days after the treatment.

The expression pattern of *FaAKR* genes in response to phytohormone ABA was also investigated. The transcriptome data from *F.* × *ananassa* cv. “Toyonoka” with exogenous ABA or NDGA treatment (Li et al., 2019) were further used to analyze. The *FaAKR23* was upregulated in response to ABA, the expression level increased by 154% on the 5th day and 26% on the 8th day (Figure 8 and Supplementary Table 7). Meanwhile, *FaAKR23* expression was suppressed in NDGA treatment and exhibited a lower expression level compared with control, which decreased by 54% on the 5th day and 72% on the 8th day (Figure 8 and Supplementary Table 7). These results suggested that *FaAKR23* might be important in ABA-mediated fruit ripening.

## Discussion

It is well-known that AsA is a key non-enzymatic antioxidant that scavenges reactive oxygen species (ROS) in plants (Akram et al., 2017). In higher plants, AsA is synthesized predominantly via the D-Man/L-Gal pathway (Wheeler et al., 1998). In general, there are higher AsA levels in green tissues such as leaves than in fruits. Because the ROS was primarily produced by photosynthesis under the light in leaves. However, some plants contain high levels of AsA in fruits, for example, strawberry (58.8 mg/100 g FW), kiwifruit (*Actinidia deliciosa*, ∼100 mg; *Actinidia eriantha*, ∼1,500 mg), acerola (*Malpighia glabra*, ∼1,700 mg), and chestnut rose (*Rosa roxburghii*, 1,000–2,000 mg) (Huang et al., 2014). Although numerous studies have focused on understanding the regulatory mechanism of AsA metabolism in model plants, how AsA content is regulated in these high AsA fruit plants is still not well-understood.

Strawberry is an important commercial horticultural crop in the world, with excellent nutritional value, attractive appearance, and unique aroma. It is well-known that strawberry fruits could accumulate plenty of AsA, which is far higher than that of the other rosaceous plants, such as apple, pear, and peach. However, the molecular mechanism of high-level AsA accumulation in strawberry ripe fruit is still unclear. The high-quality chromosome-level whole-genome sequences of diploid strawberry and octoploid-cultivated strawberry have been completed, which provides us an opportunity to carry out AsA metabolism-related gene identification and comparison analysis. In this study, we identified 125 AsA metabolism-related genes from the octoploid strawberry genome and analyzed the expression pattern using RNA-seq data. Our study provided comprehensive information on the genes for AsA biosynthesis and recycling pathway during strawberry fruit ripening.

In this study, the transcriptome data from *F.* × *ananassa* cv. “Camarosa” showed that most of the genes in the D-Man/L-Gal pathway were downregulated during fruit ripening (Figure 1). Such as, the GMP and GME were decreased from green stage to red stage, and GGP and GPP upregulated at first but then decreased (Figure 1). Similar results were also confirmed by Cruz-Rus et al. (2011) and Li et al. (2015). Furthermore, the expression of *GalLDH*, which is the final enzyme in the D-Man/L-Gal pathway, was confirmed to show no significant change among different fruit ripening stages (Cruz-Rus et al., 2011; Li et al., 2015). In another word, the D-Man/L-Gal pathway is predominant in green stage rather than in red stage. Unfortunately, no correlative sign could be found between the expression of AsA synthesis-related genes in this pathway and AsA accumulation during strawberry fruit ripening. This observation is also in accordance with previous studies on tomato fruit (Badejo et al., 2012; Lima-Silva et al., 2012) and kiwifruit (Lin et al., 2022). However, there are some fruits, such as guava (*Psidium guajava*), showing a positive correlation between gene expression level and AsA content in fruits. In particular, some genes (GME, GGP, GPP, GalDH, and GalLDH) in the D-Man/L-Gal pathway exhibited upregulated expression trends during fruit ripening, which indicates that the D-Man/L-Gal pathway is the major route for AsA biosynthesis in guava (Feng et al., 2020). The increasing data suggest that GGP is the key rate-limiting step in AsA biosynthetic pathway (Ishikawa et al., 2018). Recently, an *AceGGP3* gene was identified as the most highly expressed GGP and positively correlated with AsA accumulation in kiwifruits (*Actinidia eriantha*) (Liu et al., 2022). However, different from the AsA accumulation trend in strawberry fruit, the AsA content in kiwifruit shows a maximal AsA level at the green stage, decreased gradually with ripening, then remained fairly stable until complete ripening (Zhang J. et al., 2018; Fenech et al., 2019; Lin et al., 2022; Liu et al., 2022). Therefore, the regulation of AsA biosynthesis in the D-Man/L-Gal pathway may be different in different plants. In addition, a *cis*-acting upstream open reading frame (uORF) found in the 5’UTR region of GGP could repress the translation of the GGP under high AsA concentration in *Arabidopsis* leaves (Laing et al., 2015). A previous study found that editing the uORF of *LsGGP2* in lettuce could increase AsA content by ∼150%, indicating manipulating the translation of mRNA might also be an important regulatory pathway in AsA biosynthesis (Zhang H. et al., 2018). The feeding experiment with L-galactose in barley leaf slices, *A. thaliana* leaves, and pea embryonic axes suggested that substrate availability may also be a limiting factor of AsA biosynthesis (Wheeler et al., 1998; Gao et al., 2011).

Since the *GalUR* gene was first identified in strawberry, many studies have proved that the D-GalUA pathway played an important role in AsA biosynthesis (Agius et al., 2003; Hemavathi et al., 2009; Cai et al., 2014; Lim et al., 2016). Different from the genes in the D-Man/L-Gal pathway, the expression of *GalUR* in the D-GalUA pathway was positively correlated with the increase in AsA during strawberry fruit ripening (Agius et al., 2003; Cruz-Rus et al., 2011). In previous studies, some *GalUR* genes have been identified based on sequence alignment with *FaGalUR*. For example, the *VvGalUR* gene in grape berries (*Vitis vinifera*) was also upregulated during fruit ripening in parallel to the AsA level increment (Cruz-Rus et al., 2010). However, apart from strawberry, the encoding genes of GalUR in other plant species have not been well-studied yet (Ishikawa et al., 2018; Broad et al., 2020). Many studies found that the D-GalUA pathway seems to be predominantly species- and stage-dependent (Mellidou et al., 2021). Monosaccharides composition analysis indicates that the D-galacturonic acid was the highest represented sugar in tomato fruits (Rigano et al., 2018). The AsA content of red tomato fruits fed with D-galacturonic acid increased by 88% compared with control fruits (Badejo et al., 2012). Because D-galacturonic acid could be supplied by the breakdown of pectin in the cell wall at later stages of fruit ripening, so higher substrate availability in the D-GalUA pathway might lead to higher AsA accumulation in fruits. Therefore, this hypothesis may be explained by the reason why *GalUR* shows significantly higher expression levels along with fruit ripening. In this study, we identified four *GalUR* homologous genes, but only maker-Fvb4-1-augustus-gene-196.31 was significantly upregulated in fruit (Figure 2B).

GalUR is a member of the AKR superfamily which plays important roles in diverse plant metabolic reactions including reactive aldehyde detoxification, biosynthesis of osmolytes, secondary metabolism, and membrane transport (Sengupta et al., 2015). In addition to *FaGalUR*, whether other AKR genes in strawberry also contribute to the AsA biosynthesis remains unclear. In the present study, we further performed a genome-wide analysis of the AKR superfamily in octoploid strawberry, to identify key AKR genes participating in important physiological processes during strawberry fruit ripening. A total of 80 *FaAKR* genes were identified from the octoploid strawberry genome. The number of AKR genes varies among different plant species, such as 22 in *A. thaliana*, 29 in *Oryza sativa*, 33 in *F. vesca*, 35 in *Populus trichocarpa*, 44 in *Prunus persica*, 45 in *V. vinifera*, 49 in *Citrus clementina*, 57 in *Glycine max*, and 95 in *Malus domestica* (Duan et al., 2020). We analyzed the expression pattern of 80 *FaAKR* genes during strawberry fruit ripening. Nine genes (*FaAKR9*, *FaAKR23*, *FaAKR24*, *FaAKR27*, *FaAKR33*, *FaAKR39*, *FaAKR41*, *FaAKR49*, and *FaAKR53*) showed gradually upregulated expression (Figure 7A). We found that *FaAKR23* (maker-Fvb4-1-augustus-gene-141.33) was the most extremely upregulated *FaAKR* gene in receptacles, its expression levels increased by 142-fold from green stage to red stage (Figure 7A). More importantly, *FaAKR23* showed a close relationship with *FaGalUR* (Figures 4, 7C); therefore, it is speculated that *FaAKR23* might play an important role in strawberry fruit ripening, especially in AsA biosynthesis.

Abscisic acid plays a pivotal role in regulating the ripening and quality of strawberry fruits (Jia et al., 2011; Li et al., 2022). Exogenous ABA treatment promotes AsA accumulation, while the regulatory roles of ABA in this metabolism process have not been well-understood (Li et al., 2015, 2019). In this study, we found that most of AsA biosynthesis genes in the D-Man/L-Gal pathway were significantly downregulated in ABA-treated fruits. These results were consistent with a previous study by Li et al. (2015). Interestingly, there was also no correlation between gene expression and AsA accumulation during strawberry fruit ripening, indicating AsA accumulation might be involved in complicated regulatory mechanisms. Surprisingly, the expression of *FaAKR23*, but not *FaGalUR*, was found to have a significant correlation with ABA treatment and AsA accumulation. Furthermore, *FaAKR23* has the highest expression level in the AKR superfamily during fruit ripening, and it is hypothesized that this gene might be involved in ABA-mediated AsA biosynthesis. In future, further studies should be carried out to verify whether *FaAKR23* is involved in ABA-mediated AsA biosynthesis in strawberry.

## Conclusion

In this study, we first identified 125 AsA metabolism-related genes in the octoploid strawberry genome and analyzed gene expression patterns using two available RNA-seq data. The results showed that the *FaGalUR*, the D-galacturonate reductase gene in the D-GalUA pathway, was extremely upregulated in receptacles during strawberry fruit ripening but hardly expressed in achenes, leaves, and roots. We further implemented a genome-wide analysis of the *FaAKR* genes and identified a putative key gene, *FaAKR23*, which showed a higher expression level than *FaGalUR* in receptacles, and was confirmed by qRT-PCR analysis. Furthermore, the *FaAKR23* showed a positive role in AsA and ABA accumulation, indicating its important role in ABA-mediated strawberry fruit ripening. Our study provides useful information on the AsA metabolism during strawberry fruit ripening and will help understand the mechanism of AsA accumulation in strawberry fruits.

## Data Availability Statement

The original contributions presented in the study are included in the article/Supplementary Material, further inquiries can be directed to the corresponding authors.

## Author Contributions

HL performed the experiments, analyzed the data, and wrote the manuscript. LW and YN assisted in the experiments. HL and YG conceived the project. All authors revised and contributed to the manuscript and approved the submitted version.

## Conflict of Interest

The authors declare that the research was conducted in the absence of any commercial or financial relationships that could be construed as a potential conflict of interest.

## Publisher’s Note

All claims expressed in this article are solely those of the authors and do not necessarily represent those of their affiliated organizations, or those of the publisher, the editors and the reviewers. Any product that may be evaluated in this article, or claim that may be made by its manufacturer, is not guaranteed or endorsed by the publisher.

## Footnotes

1
https://www.arabidopsis.org/index.jsp

2
https://www.rosaceae.org/species/fragaria_x_ananassa/genome_v1.0.a1

3
http://pfam.xfam.org/

4
https://www.ncbi.nlm.nih.gov/cdd/

5
https://www.ebi.ac.uk/Tools/pfa/pfamscan/

6
http://smart.embl-heidelberg.de/

7
http://www.expasy.org

8
http://bioinformatics.psb.ugent.be/webtools/plantcare/html/
