# Supplementary material for: Genome-Wide Analysis of Ascorbic Acid Metabolism Related Genes in Fragaria × ananassa and Its Expression Pattern Analysis in Strawberry Fruits
Source: Front Plant Sci. 2022 Jul 6;13:954505. doi: 10.3389/fpls.2022.954505 (PMC9296770; doi:10.3389/fpls.2022.954505)
Supplement: Supplementary file 1 [file Table_1.DOCX]

**Supplementary Table 1** The primers used for qRT-PCR analysis.

| gene | orientation | sequence |
| --- | --- | --- |
| *FaAKR23* | Forward | CGCAAAATGATGCCTCGAGAA |
|  | Reverse | ACATGAGTAGCTGACGCCTTT |
| *FaAKR24* | Forward | CTTCCACAGCGGAAAGGGGT |
|  | Reverse | TATCCCGGCGGTAAAATTGG |
| *FaCHP1*  (reference) | Forward | TGCATATATCAAGCAACTTTACACTGA |
|  | Reverse | ATAGCTGAGATGGATCTTCCTGTGA |
